# A Review of Oxylipins in Alzheimer’s Disease and Related Dementias (ADRD): Potential Therapeutic Targets for the Modulation of Vascular Tone and Inflammation

**DOI:** 10.3390/metabo12090826

**Published:** 2022-09-01

**Authors:** Lynne H. Shinto, Jacob Raber, Anusha Mishra, Natalie Roese, Lisa C. Silbert

**Affiliations:** 1 Department of Neurology, Oregon Health & Science University, 3181 SW Sam Jackson Park Rd., CR120, Portland, OR 97239, USA; 2 Departments of Behavioral Neuroscience and Radiation Medicine, Division of Neuroscience, ONPRC, Oregon Health & Science University, Portland, OR 97239, USA; 3 Jungers Center for Neurosciences Research, Oregon Health & Science University, Portland, OR 97239, USA; 4 Veterans Affairs Medical Center, Portland, OR 97239, USA

**Keywords:** review, oxylipin, fatty acids, vascular dementia, Alzheimer’s disease

## Abstract

There is now a convincing body of evidence from observational studies that the majority of modifiable Alzheimer’s disease and related dementia (ADRD) risk factors are vascular in nature. In addition, the co-existence of cerebrovascular disease with AD is more common than AD alone, and conditions resulting in brain ischemia likely promote detrimental effects of AD pathology. Oxylipins are a class of bioactive lipid mediators derived from the oxidation of long-chain polyunsaturated fatty acids (PUFAs) which act as modulators of both vascular tone and inflammation. In vascular cognitive impairment (VCI), there is emerging evidence that oxylipins may have both protective and detrimental effects on brain structure, cognitive performance, and disease progression. In this review, we focus on oxylipin relationships with vascular and inflammatory risk factors in human studies and animal models pertinent to ADRD. In addition, we discuss future research directions with the potential to impact the trajectory of ADRD risk and disease progression.

## 1. Introduction

There are currently an estimated 55 million individuals with Alzheimer’s disease and related dementias (ADRD) worldwide, and this figure is projected to increase to 78 million by 2030, coincident with a growing global elderly population [1]. ADRD contributes to a diminished quality of life for patients and caregivers, and results in significant individual and societal financial burdens. Cerebrovascular disease (CVD) is one of the largest single identifiable risk factor for dementia apart from age, and one of the few that is potentially preventable [2]. This is made all the more important in light of evidence that combinations of different co-pathologies are more important in predicting cognitive impairment than any single pathology [3,4,5]. In particular, mixed AD and vascular pathologies increase one’s risk for dementia nearly two-fold compared with those with AD pathology alone [3]. Vascular cognitive impairment (VCI) and AD share common risks factors [6,7,8,9], and post-mortem studies demonstrate correlations between the presence of AD and CVD pathologies [10,11,12]. Potential cerebrovascular contributions to ADRD include diminished cerebral perfusion, inflammation, and impaired clearance of toxic solutes, including amyloid beta and tau, involving paravascular drainage pathways [10,12,13]. In addition, ADRD risk factors include diseases of the peripheral cardiovascular system, such as hypertension, atherosclerosis, and coronary artery disease (CAD), as well as conditions that alter peripheral vascular beds and blood flow, such as diabetes [1].

It is recognized that even modest delays in dementia onset at the individual level could have a significant impact on dementia prevention on a global scale [13]. There is, therefore, an urgent need for accessible means, including dietary modifications, by which individuals may optimize their vascular health. A diet rich in omega-3 fatty polyunsaturated fatty acids (PUFAs) is thought to be neuroprotective, and is recommended for primary and secondary stroke prevention [14,15]. However, the exact mechanisms by which omega-3 fatty acids confer beneficial effects on brain health are not well defined, and results from epidemiological studies and clinical trials aimed at preserving cognitive function in aging and AD have shown mixed results. Diets with a higher omega-6/omega-3 fatty acid ratio have been shown to promote ADRD risk factors, such as adiposity and inflammation [16,17]; however, little is known about the downstream PUFA metabolites that may be driving these relationships. Oxylipins are a class of bioactive lipid mediators derived from the metabolism of long-chain omega-3 and omega-6 polyunsaturated fatty acids (PUFAs) that act as modulators of both vascular tone and inflammation. There is emerging evidence that PUFA-derived oxylipins may confer both positive and detrimental effects. In this review, we focus on the relationships between PUFA-derived oxylipins and ADRD risk with an emphasis on CVD. The overall goal of this review is to highlight the potential mechanisms by which PUFA-derived oxylipins may serve as targets for interventions aimed at preserving cognitive function in older individuals.

## 2. Long-Chain PUFAs

**Figure 1:**
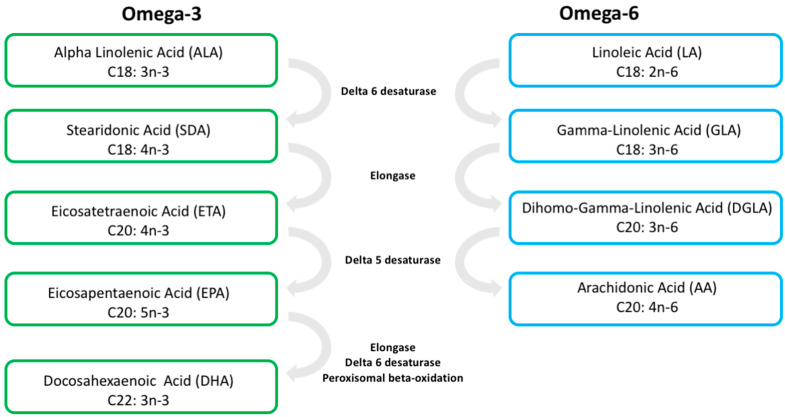
The formation of long-chain polyunsaturated fatty acids involve the addition of double bonds by Delta 5 and Delta 6 desaturase and the elongation of the carbon chain by Elongase. Arachidonic acid (AA), Eicosapentaenoic acid (EPA), and Docosahexaenoic acid (DHA) are synthesized by a stepwise process that starts with the desaturation of the 18-carbon essential fatty acids Alpha Linolenic Acid (ALA) and Linoleic Acid (LA). Delta-5 and -6 desaturases are rate-limiting enzymes; thus, there is a competitive relationship between ALA and LA in the formation of AA, EPA, and DHA.

The two main classes of long-chain PUFAs are the omega-3 (n-3) and omega-6 (n-6), named for the carbon position of the double bond away from the terminal methyl group, omega-3 at carbon 3 and omega-6 at carbon 6 [18] (Figure 1). The 18-carbon n-3 alpha linolenic acid (ALA), and the 18-carbon n-6 linoleic acid (LA) are essential PUFAs and must be obtained through diet, because humans and other mammals cannot make these fatty acids from smaller carbon chain PUFAs [18]. The longer-chain n-3 PUFAs, eicosapentaenoic acid (EPA) and docosahexaenoic acid (DHA), and n-6 PUFA arachidonic acid (AA) are synthesized from ALA (n-3) and LA (n-6) through a series of enzymatic steps that elongate the fatty acid chain with the addition of double bonds. The addition of double bonds by delta-5 and delta-6 desaturase is a rate-limiting step; therefore, ALA (n-3) and LA (n-6) have a competitive relationship in the formation of EPA, DHA, and AA [19,20] (Figure 1).

Due to this competitive relationship with limited desaturase enzymes, the ratio of n-3 and n-6 obtained through diet or dietary supplementation can drive the synthesis towards the production of a higher or lower n-3:n-6 ratio in cells [19,20]. The consumption of a standard Western diet high in animal fat, fried and processed foods, and low in whole grains, fruits, and vegetables has shifted the n3:n6 ratio from a heathier 1:4 ratio, with observed decreases in cardiovascular disease and mortality, to an unhealthy 1:20 ratio [17,18,19,20,21,22]. Plant sources contain both n-3 and n-6 essential fatty acids, with nuts and seeds containing a higher ratio of LA:ALA, while still maintaining a healthy ratio of approximately 1:4 n-3:n6 [23]. Chia and flaxseeds and green leafy vegetables contain a higher ratio of ALA:LA, whereas corn and safflower oils contain very high levels of LA [19]. This is to highlight that foods in the diet are the main determinants to a healthy n-3:n-6 balance [17,18,20,24]. Dietary sources of EPA and DHA include cold-water fish (e.g., salmon, mackerel, and sardine); dietary sources of AA include red meat, fats, butter, and egg yolks [19]. Long-chain PUFAs, EPA, DHA, and AA, have two main functions in cells: (1) as phosopholipids, they contribute to cell membrane fluidity; and (2) after enzymatic cleavage from the cell membrane, PUFAs become oxidized to bioactive free oxylipins with multiple functions that include the modulation of inflammation, vasodilation and constriction, and cell proliferation [19,25,26].

## 3. Oxylipins

**Figure 2:**
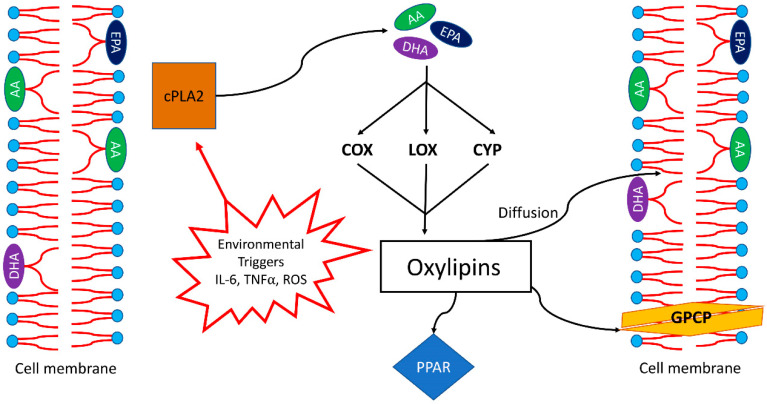
Formation of oxylipins from PUFAs in the membrane. In inflammation, reactive oxygen species (ROS) activate cytosolic phospholipase A2 (cPLA2), releasing PUFAs from the cell membrane. PUFAs are rapidly oxidized by COX, LOX, and CYP enzymes to form bioactive oxylipins. Oxylipins exert effects by diffusion, binding to membrane receptors (e.g., G-protein-coupled receptors (GPCP), and binding to peroxisome proliferator-activated receptors (PPARs) in the nucleus and cytosol. AA, arachidonic acid; EPA, eicosapentaenoic acid; DHA, docosahexaenoic acid; IL-6, interleukin 6; TNFα, tumor necrosis factor alpha; COX, cyclooxygenase; LOX, lipoxygenase; CYP, cytochrome P450. For details, see the main text.

Oxylipins are a broad class of bioactive metabolites formed from the enzymatic and non-enzymatic oxidation of fatty acids. The n-3 class of parent PUFAs are ALA, EPA, and DHA, and the n-6 class of parent PUFAs are LA and AA [25,26]. PUFAs are an integral part of all cell membranes. In the brain, DHA and AA constitute 25% of the total fatty acids in neuronal membranes and contribute to membrane structure and fluidity [26]. Most PUFAs are sequestered as phospholipids in the cell membrane to control the formation of oxylipins [26]. When PUFAs are released from the membrane, they remain in a free fatty acid form very briefly before they are rapidly oxidized and transformed into bioactive oxylipins, which play multifaceted roles in cell signaling, affecting intra- and intercellular metabolism and regulation [25,26,27]. These signaling roles become particularly pronounced upon cell stress and/or injury, which activates cytosolic phospholipase A2 (cPLA2) to cleave specific PUFAs from the membrane producing free fatty acids, with downstream effects on inflammatory markers (e.g., tumor necrosis factor alpha (TNFα) and interleukin-6 (IL-6)), reactive oxygen species (ROS), and calcium (Ca^2+^) signaling. Intriguingly, amyloid beta oligomers can also trigger cPLA2 activation [25,27] (Figure 2).

There are over 50 subspecies of PLA2, some that cleave specific PUFAs. In the brain, PLA2 is found in both neurons and glial cells [27]. Once released into the cytosol, PUFAs are oxidized by cyclooxygenase (COX), lipoxygenase (LOX), and cytochrome P450 (CYP) enzymes to form bioactive oxylipins that exert their effects by diffusion through membranes, binding to membrane receptors (e.g., G-protein-coupled receptors), or binding to peroxisome proliferator-activated receptors (PPARs) in the nucleus and in the cytosol [25,26,27] (Figure 2). Diet impacts the ratio of n3:n6 PUFAs incorporated into cell membranes; consequently, it has an influential role in the ratio of n3:n6-derived oxylipins produced in a variety of tissues, including the brain. In mouse models, diets supplemented with n-6 linoleic acid (LA) show an increase in LA-derived oxylipins in the brain, whereas diets supplemented with the n-3 fatty acids, EPA and DHA, show an increase in n-3-derived oxylipins in plasma, brain, and colon [28,29].

**Figure 3:**
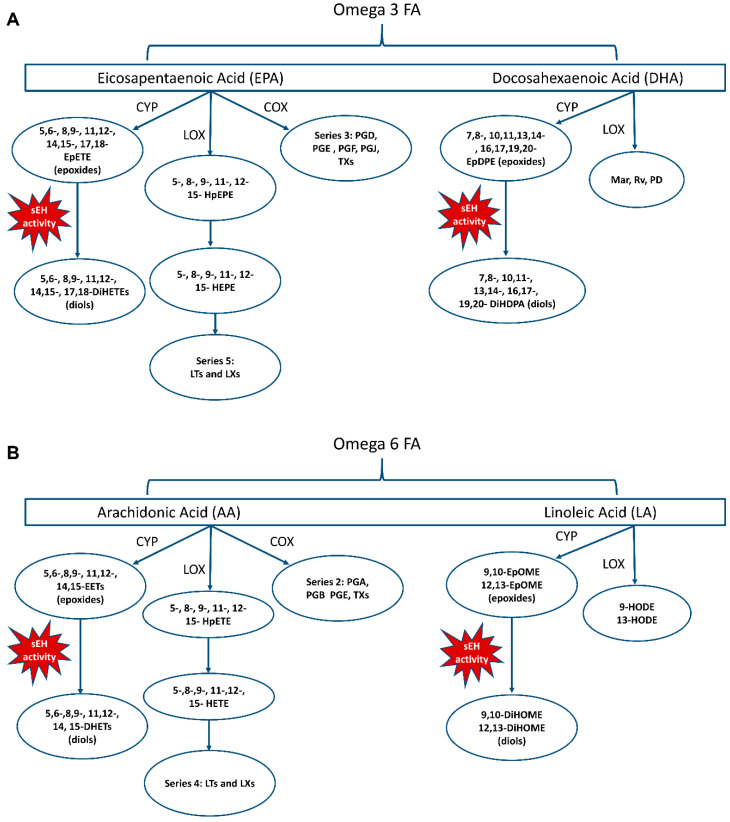
(**A**) Enzymatic oxidation of n-3 PUFAs forming oxylipins. n-3: EPA, eicosapentaenoic acid; DHA, docosahexaenoic acid. (**B**) Enzymatic oxidation of n-6 PUFAs forming oxylipins. N-6: AA, arachidonic acid; LA, linoleic acid. COX, cyclooxygenase; CYP, cytochrome P450; sEH, soluble epoxide hydrolase, which converts epoxides to diols. See Supplementary Table S1, Select Oxylipin Nomenclature, for the full names of oxylipin abbreviations.

COX transforms AA and EPA into prostanoids that include series 2 prostaglandin A_2_, B_2_, D_2_, and E_2_, and thromboxane A_2_, and series 3 prostaglandin D_3_, E_3_, F_3_, and J_3_, and thromboxane A_3_ (Figure 3A,B, Supplementary Table S1). The COX-derived prostaglandins are inflammatory and vascular tone mediators, whereas thromboxanes affect vascular function by increasing platelet aggregation, contributing to vessel occlusion [25,26,30]. LOX transforms PUFAs into hydroxy fatty acids that are further metabolized to AA-derived hydroxy-eicosatetraenoic acids (HETEs) and series 4 Leukotrienes (LT); LA-derived hydroxy-octadecadienoic acids (HODEs); EPA-derived hydroxy-eicosapentaenoic acids (HEPEs) and series 5 LT; DHA-derived maresins (Mars), protectins (PDs), and resolvins (Rvs) [25,26,31] (Figure 3A,B, Supplementary Table S1). Cytochrome P450 (CYP) is named for its absorbance band at 450 nm and is a large family of heme-containing monooxygenases that oxidize a wide variety of compounds [25,26]. CYP2C and CYP2J have expoxygenase activity and will convert PUFAs to epoxides that are further metabolized by the enzyme-soluble epoxide hydrolase (sEH) to diols [32,33] (Figure 3A,B, Supplementary Table S1). AA-derived epoxides are called epoxy-eicosatrienoic acids (EETs), LA-derived epoxides are called epoxy-octadecenoic acids (EpOMEs), EPA-derived epoxides are called epoxy-eicosatetraenoic acids (EpETEs), and DHA-derived epoxides are called epoxy-docosapentaenoic acids (EpDPEs). Their corresponding diols are, for AA, dihydroxy-eicosatetraenoic acids (DHET); for LA, dihydroxy-octadecenoic acids (DiHOMEs); for EPA, dihydroxy-eicosatetraenoic acids (DiHETEs); and for DHA, dihydroxy-docasapentaenoic acids (DiHDPAs) (Supplementary Table S1).

Soluble epoxide hydrolase (sEH) hydrolyzes epoxides, converting them to their corresponding diols. In humans, sEH is found in many tissues, including the liver, kidney, vascular smooth muscle, pituitary gland, pancreas, and muscle [34], and in the brain it is found in neuronal cell bodies, oligodendrocytes, and astrocytes [35]. CYP-derived epoxides have been shown to exert anti-inflammatory and vasodilatory effects that are diminished when converted to diols [32,33,36,37]. Studies testing the effects of sEH inhibitors showed that blocking sEH activity increased the epoxy-to-diol ratio with robust positive outcomes. This has been well studied in hypertension, where blocking the conversion of AA-derived EETs to DHETs using specific sEH inhibitors in animal and human studies had a significant effect on lowering blood pressure [32,33,36,37]. In general, converting an epoxide to its corresponding diol weakens the epoxide effect. Epoxide hydrolase 2 (EPHX2) is the gene that produces sEH and there are at least 26 EPHX2 snps that have been identified, 2 that have been associated with sEH activity. K55R is an EPHX2 snp that increases sEH activity and is associated with hypertension, cardiovascular disease, and ischemic stroke in human populations [33,38]. The R287Q EPHX2 snp decreases sEH activity and, although its effect on health outcomes is less clear than K55R in human populations, in vitro studies of ischemic injury show enhanced neuronal survival linked to R287Q [33,38]. There is also emerging evidence in human studies that Apolipoprotein E4 (E4) carriers may have higher sEH activity compared with homozygous E3 carriers [39]. In a report by Saleh et al., healthy controls who were either homozygous E3 carriers or heterozygous/homozygous E4 carriers were given the same dose of EPA + DHA supplementation over 12 months. As expected, EPA and DHA levels significantly increased at 12 months in both groups, and there was no difference between E4 carriers and non-carriers in EPA and DHA levels. At 12 months, however, E4 carriers had significantly higher diol-to-epoxide ratios of EPA and DHA oxylipins than E3 carriers [39]. Taken together, these findings support the presence of genetic effects involving EPHX2 and ApoE isoform on sEH activity that may be relevant to n-3 PUFA effects on ADRD. This genetic heterogeneity could at least partially account for the discrepancies in PUFA efficacy in AD between animal and human studies and differences in treatment effects between E4 carriers and non-carriers in previous human clinical trials [40].

Well-studied subclasses of oxylipins include eicosanoids that include prostaglandins, thromboxanes, and leukotrienes derived from AA, and docosanoids that include maresins, protectins, and resolvins derived from DHA [41,42,43]. Several excellent reviews have been published on AA oxylipins (HETEs, EETS, DHETS, and PGs) in animal and human studies of hypertension, cardiovascular disease, and stroke [32,36,37,44,45,46,47,48], and in ADRD [30,49,50,51,52], as well as on the DHA LOX-derived maresins, protectins, and resolvins in ADRD [41,42,53]. This review will focus on less well-studied oxylipins that include LA LOX-derived HODES and CYP-derived EpOMEs and DiHOMEs, EPA LOX-derived HEPES and CYP-derived EpETEs, DiHETEs, and DHA CYP-derived EpDPEs and DiHDPAs, highlighting the existing research on their roles in vascular risk factors and ADRD.

## 4. n-3 PUFAs and Oxylipins: Animal Studies

Animal studies investigating the influence of dietary PUFA intake suggest that they have strong effects on multiple aspects of brain function, with potential implications for conditions that affect neuroinflammation, vascular dysfunction, and neurodegeneration [54]. Increasing the dietary intake of n-3 PUFAs has been shown to decrease infarct burden, reduce mortality, and enhance the likelihood of reperfusion without intervention after ischemic insults in mice [55]. Furthermore, n-3 PUFAs appear to alleviate behavioral alterations and cognitive injury in several assays, suggesting a positive impact on learning and memory [56]. In a study performed in stroke-prone spontaneously hypertensive rats, increasing the DHA intake suppressed the age-dependent development of hypertension, prolonged life span, and increased behavioral and cognitive performance, while having no effect on infarct burden or histopathological changes [57]. In another study, increasing the n-3:n-6 ratio in the chow of rats for several months before exposing them to a brief global ischemia improved spatial memory and alleviated microvascular dysfunction, as shown by increased pericyte density [58].

Overall, these findings suggest that n-3 PUFAs such as DHA alleviate functional deterioration not only by improving vascular risk factors, but may even be protective against the detrimental effects of acute cerebrovascular incidents such as ischemia. Such beneficial effects of n-3 PUFAs are largely mediated by their metabolism into various oxylipins [25]. However, a comprehensive picture of how the different oxylipins affect specific cellular and molecular pathways to alter brain function is not yet in focus. A case in point is the role of soluble epoxide hydrolase (sEH), an enzyme that can produce a subset of oxylipins, which may not always be favorable. For example, sEH metabolizes DHA to 19,20-dihydroxydocosapentaenoic acid (19, 20-DiHDPA), which alters membrane lipid composition in a way that disengages intercellular interactions between pericytes and endothelial cells as well as between endothelial cells, ultimately resulting in increased blood–brain barrier permeability and pericyte loss in a model of diabetic retinopathy [59]. A stark increase in sEH expression accompanies the development of retinopathy in diabetic humans, suggesting that conservation of these mechanisms may indeed underlie the human disease [59]. Stroke studies using animal models have found that pharmacologically inhibiting or genetically eliminating sEH activity has mixed effects on infarct size but enhances post-reperfusion cerebral blood flow [60] and may be protective against infarcts [61]. However, in another study, inserting a human variant of sEH that more preferentially translocates to peroxisomes in mice decreased ischemia-induced injury [62], suggesting a complex role of sEH in cerebrovascular function depending on its sub-cellular organelle-level localization, perhaps due to its actions on, or metabolism of, different subsets of PUFA oxylipins.

Due to the close association of cerebrovascular dysfunction and ADRD, these findings open up the possibility that increasing dietary n-3 PUFAs, perhaps in combination with decreasing n-6 PUFAs, could slow dementia-related pathogenesis and cognitive decline. In an effort to test this concept, one recent study used the senescence-accelerated mouse-prone 8 (SAMP8) model of dementia and examined the effect of feeding the mice a diet high in DHA or green nut oil, which contains high amounts of the DHA precursor ALA [63]. They found that both dietary approaches improved hippocampus-dependent spontaneous alternation in the Y-maze task, while concurrently raising brain EPA and DHA levels [63]. Multiple studies have also tested the effects of dietary n-3 PUFAs on AD pathogenesis itself, in mice injected intracerebroventricularly with amyloid beta peptide 25–35 (Aβ25–35 peptide), which exhibited a marked improvement in learning and memory [64,65,66]. Multiple mechanisms have been implicated in these beneficial benefits bestowed by n-3 PUFAs. In one study, n-3 PUFAs were found to lower the amyloidogenic pathway by downregulating beta-secretase 1 (BACE-1) and Presenilin-2 (PS2) expression, while enhancing their breakdown by upregulating A Disintegrin and Metalloproteinase 10 (ADAM10), soluble amyloid precursor protein alpha (sAPPα), and c-terminal fragments (CTFs) [65]. In another study, n-3 PUFAs downregulated inflammatory signal generators, such as inducible nitric oxide synthase and COX-2, while enhancing survival signals such as brain-derived neurotrophic factor (BDNF) [64]. In yet another study, n-3 PUFAs increased the antioxidant activity of glutathione peroxidase and superoxide dismutase [66]. Furthermore, this latter study reported improvements in memory and decreases in phosphorylated tau in a dose-dependent manner [66], which hold promising implications for n-3 PUFAs in ADRD. A wealth of evidence exists to suggest a role of n-3 PUFAs in promoting neurite growth, enhancing synaptic function, reducing pro-inflammatory signals while enhancing anti-inflammatory signals (e.g., neuroprotection D), and preventing neuronal apoptosis (reviewed in detail in [67]). Due to the impressively broad array of bioactive oxylipins synthesized from n-3 PUFAs [25], it is possible that distinct downstream oxylipin signals may enact these wide-ranging effects of n-3 PUFAs and contribute to their overall beneficial effects on brain function.

## 5. n-3 PUFAs and Brain Fatty Acid Composition: Animal Studies

Notably, levels of n-3 PUFAs EPA and DHA rose in the brain not only after supplementing the diet directly with these molecules, but also when supplementing the diet with the n-3 essential fatty acid, alpha linolenic acid (ALA), or plant oils rich in ALA [63,64,65,68]. These results are not surprising because ALA is a precursor to both n-3 PUFAs. EPA is generated from ALA by the sequential actions of delta-6 desaturase, elongase, and delta-5 desaturase, while DHA is then generated from EPA via elongase and delta-6 desaturase via a peroxisomal beta-oxidation reaction (Figure 1). Indeed, it appears that, at least in rats, brain accretion of DHA is lower than that of ALA following dietary supplementation, although ALA is just as efficient in increasing brain DHA levels, suggesting that local metabolism may be the primary source of DHA within the brain [68]. In accordance, intervention studies that employ increased trans-fatty acids in the diet to bias metabolism towards n-6 PUFAs show no change in brain DHA levels, despite reducing DHA levels in most other organ systems, suggesting compensatory de novo synthesis in the brain [69]. These studies are supported by findings that cultured astrocytes—the primary glial cells of the brain—constitutively produce DHA from several precursors including ALA [70] and that astrocyte-generated DHA can be shuttled and incorporated into neurons [70]. Brain endothelial cells have also been shown to elongate and desaturate PUFAs, preferentially n-3, to synthesize DHA [71], which could function to increase brain EPA and DHA levels as an integral aspect of PUFA uptake across the blood–brain barrier. Nonetheless, changes in lipid composition outside the brain, either on other organ systems or on the cardiovascular system, may still indirectly impact brain function. Two specific examples of such indirect effects are the gut microbiome and the gut–liver–brain axis, as described subsequently.

Although the primary source of DHA is still believed to be dietary, these animal studies suggest that ALA uptake and downstream metabolism may be equally important mechanisms regulating DHA levels in the brain. Considering that ALA is accreted by the brain at a higher rate compared with DHA, supplementation with dietary ALA products may be more effective in increasing brain n-3 PUFAs and capitalizing on their protective effects against vascular dysfunction and neurodegeneration. This may prove to be a more sustainable, equitable, and broadly applicable solution (e.g., for individuals who are vegan or face harsher socioeconomic situations), because ALA can be found in high amounts in certain plant-based dietary sources as compared with EPA or DHA, which are primarily enriched in animal-based products, mainly fish and seafood. Future studies examining whether ALA uptake and metabolism to EPA/DHA are just as effective in human populations are necessary to support these suggestions.

## 6. Oxylipins and the Gut Microbiome: Animal Studies

As discussed previously, in some conditions, there might be differential effects of diet on oxylipins in and outside the brain. It is important to recognize that in addition to direct and indirect effects mediated by vascular changes in and outside the brain, there might be indirect effects of diets on oxylipins mediated by alterations in the gut microbiome and mediated by the gut–liver–brain axis [72]. In humans, gut microbiomes diversify with age, reflect healthy aging, and predict survival [73]. Growing evidence also links alterations in the gut microbiome to the development of neurodegenerative conditions, including Parkinson disease [74,75] and Alzheimer’s disease [76,77].

The gut microbiome plays an important role in the pathology of obesity [78,79] and metabolic disorders related to inflammation [80]. Gut microbiota can either protect by being anti-inflammatory or cause injury by being pro-inflammatory [81]. Alterations in the composition of the gut microbiome, induced by administering wide-spectrum antibiotics (dysbiosis) in the drinking water for two weeks, altered oxylipins in the plasma of young adult male Wistar rats on a standard chow diet (kcal/100 g: 72.4% carbohydrate, 8.4% lipid, and 19.3% protein; Safe-A04c, Germany) or a cafeteria diet composed of highly palatable and energy-dense human foods (kcal/100 g: 58.2% carbohydrate, 31.1% lipid, and 10.7% protein) [82]. In rats on standard chow, gut microbiota dysbiosis caused by wide-spectrum antibiotics did not change the overall plasma oxylipin profile, but increased 4-HDHA and 8-HEPE, related to anti-inflammatory effects, and decreased 15(R)-Lipoxin A4/A5 levels, related to pro-resolving effects. In rats on the cafeteria diet, there was an overall increase in the abundance of pro-inflammatory bacteria and a reduction in plasma oxylipin levels, involving both pro- and anti-inflammatory metabolites.

This pattern of reduced plasma oxylipin levels is consistent with what has been observed in soybean-oil-induced obese mice [83]. There is some evidence that gut microbiota affect plasma oxylipins under conditions of diet-induced obesity. In rats on the cafeteria diet treated with wide-spectrum antibiotics, there were increases in pro- and anti-inflammatory plasma oxylipin metabolites compared with diet-matched rats that did not receive wide-spectrum antibiotics. When the relationship between the abundance of gut microbiota and plasma oxylipins was assessed, gut microbiota mainly affected by wide-spectrum antibiotics were correlated with plasma oxylipin levels. Bacteroidetes showed negative correlations with most of the plasma oxylipins, including 16-HDHA, 8-HEPE, LTB4, and PGD2. Oxylipins 16-HDHA and 8-HEPE are derived from the anti-inflammatory n-3-PUFAs, DHA and EPA, whereas LTB4 and PGD2 are derived from the pro-inflammatory n-6-PUFA, AA. Proteobacteria showed both negative and positive correlations, including a positive correlation with the pro-inflammatory oxylipin LTB4 [84].

There is also evidence for a role of the gut microbiome in mediating the effects of diet on the brain involving the gut–liver–brain axis in neuropsychiatric conditions, including depression (for a review, see [85]). Depression is a common symptom in ADRD, and depression on its own contributes to poorer cognitive performance [86]. Consistent with this notion, a diet including nopal cactus, soy protein, turmeric, and chia seed oil decreased high-fat-diet-induced alterations in the liver and brain while improving cognitive performance in young adult male Wistar rats. Parts of nopal cactus and chia seeds have a high n3:n6 ratio that may contribute to the effects are associated with restoration of the gut microbiome, improved cognitive performance, and decreased neuroinflammation [87].

## 7. n-PUFAs in ADRD: Human Studies

Supplementation of EPA has been shown to decrease pro-inflammatory compounds, such as tumor necrosis factor alpha (TNF-α), interleukin 1 (IL-1), and prostaglandin E-2, in both animal and human studies [88]. The most recent meta-analyses evaluating human randomized double-blind, placebo-controlled trials (RCTs) of n-3 supplementation in cardiovascular disease report a mild to modest benefit in decreasing the risk of major cardiovascular events, cardiovascular disease-related death, and myocardial infarction [89,90]; RCTs evaluating n-3 supplementation for blood pressure showed a mild effect on reducing systolic and diastolic pressure in people who were normotensive and hypertensive [91]. These findings suggest that an imbalance between n-3 and n-6 PUFAs, and their downstream bioactive oxylipins, could be a contributing factor in these clinical conditions.

DHA and AA are the most abundant PUFAs in the brain, making up 25% of the phospholipids in grey matter. AA plays a critical role during the early stages of human brain development [92,93], and AA metabolites regulate cerebrovascular tone and neurovascular coupling [94,95]. However, AA’s protective role in brain aging and dementia is less clear [93]. For dementia risk, longitudinal epidemiological studies in healthy, non-demented, middle-aged to older adults showed that people who eat fish at least once a week versus people who eat fish less frequently or not all had a 40–60% decreased risk of dementia and AD [96,97,98,99]. On the other hand, two of the larger RCTs evaluating supplementation with both EPA and DHA, or DHA alone, found no difference between a placebo and n-3 supplementation on changes in cognitive performance, and activities of daily function in people with mild to moderate AD [40,100]. The two studies include Freund-Levi et al., who enrolled 174 participants and supplemented them with 1.7 g DHA plus 0.60 g EPA over 6 months [100], and Quinn et al., who enrolled 402 participants and supplemented them with 2.0 g DHA over 18 months [40]. Andrieu et al. conducted a four-arm dementia prevention study in 381 participants with memory complaints who were not demented that evaluated DHA (0.80 g) plus EPA (0.225 g), 340 participants with the same dose of omega-3 fatty acids combined with training in diet, exercise, and cognitive skills, 380 participants with the training without omega-3 fatty acids, and 380 participants in a placebo treatment group. The 3-year intervention showed no significant difference in cognitive performance in any of the treatment arms compared with placebo [101]. The mismatch in outcomes between the epidemiological n-3 studies on dementia risk and RCT n-3 supplementation studies for dementia warrants further exploration. Confounding factors could include the duration of supplementation or even bioavailability, absorbance, and/or the brain accretion of lipids from the different sources. A better understanding of bioactive PUFA metabolites, and specific oxylipin effects, on inflammation and vascular tone may help to elucidate the mechanism by which n-3 PUFAs exert their effects in ADRD, whether they are obtained through diet or through supplementation.

## 8. Specific Oxylipins Associated with Vascular Risk Factors

### 8.1. Linoleic Acid—Omega-6-Derived Oxylipins: HODEs, EpOMES, and DiHOMES

LA can cross the blood–brain barrier and enters the brain at approximately the same rate as AA and DHA; however, unlike AA and DHA, only a small percentage of LA is incorporated into brain phosopholipid membranes [24,102]. The majority of LA is converted to polar compounds, including oxylipins, by beta-oxidation, and by enzymatic (LOX, COX, and CYP) and non-enzymatic oxidation [24,102]. In rodent studies, an LA-enriched diet increased LA levels in plasma, and also in the cerebrum and cerebellum [29]. In addition, LA-derived oxylipins, including 9,13-HODE, 9,10-, 12,13-EpOME and their corresponding sEH-dervied diols, are increased in the cortex. LA-derived CYP oxylipins are presented in CYP and sEH sections.

### 8.2. HODES

In human and animal atherosclerotic plaques, 9- and 13-HODE are the most prevalent oxylipins. In a rabbit model of atherosclerosis, both HODEs were two of the five oxylipins with the highest concentration in aortic plaques [103]. HODES are produced enzymatically by LOX and non-enzymatically by reactive oxygen species (ROS, e.g., peroxides and superoxide), and can be synthesized by macrophages, vascular smooth muscle cells, endothelial cells, and platelets [104,105,106]. Studies that have evaluated HODEs from atheromas in people undergoing carotid endarterectomy have measured specific stereoisomers of 9- and 13-HODE that are formed by LOX or non-enzymatically by reactive oxygen species [105,107,108,109]. In general, 9-HODE is thought to possess proinflammatory effects, contributing to the progression of atherosclerotic plaques, whereas 13-HODE is thought to possess anti-inflammatory and anti-thrombotic effects and play a protective role in atherosclerosis [25,104]. In the early formation of human plaques, LOX predominantly forms S- and R-13-HODE with higher ratios of S:R and 13-HODE, but little or no 9-HODE is present. In advanced plaques, equal ratios of S- to R-13-HODE and equal ratios of 9- to 13-HODE have been measured. These HODEs appear to be formed non-enzymatically, reflecting the milieu of higher inflammation and oxidative stress with plaques at this stage [108]. The stereoisomer forms of HODE have been reported to have different and sometimes opposing actions [104]: S-13-HODE is the major oxygenation product from low-density lipoprotein (LDL) identified in atherosclerotic tissue samples [108], whereas non-enzymatically formed R-13-HODE is present in vascular endothelial cells, vascular smooth muscle cells, and at higher density in macrophage-enriched atherosclerotic plaques, and is thought to be more atherogenic than the S-13-HODE form [105]. In one study, R-9-HODE increased natural-killer-cell-secreted interferon gamma, whereas S-9-HODE decreased it [104]. In a study designed to evaluate the relationship between HETEs and HODEs with atherosclerotic plaque instability (histopathology) and patient symptoms (including transient ischemic attack and stroke versus asymptomatic), all plaque types exhibited the non-enzymatic formation of 9-/13-HODEs and AA-derived 15-/11-HETEs with no difference in oxylipins association with histopathology or symptoms [109]. In another study that evaluated oxylipin differences in people with ischemic stroke (*N* = 75) compared with controls (*N* = 35), 9-/13-HODE levels were lower and LOX-derived RvD1 from DHA was lower in the ischemic stroke group. The authors conclude that 9-/13-HODE along with RvD1 may be important regulators of inflammation in stroke [110]. Another prospective study evaluating HODEs in children (mean age 2.5 (range 0.60–12.0 years) undergoing cardiopulmonary bypass surgery (CPB) found that there was no change in LA from the start to end of surgery, whereas 9- and 13-HODE levels increased at the beginning of surgery. There was no association between individual HODEs with markers of morbidity and mortality measured by vasoactive inotropic score (VIS) or with milirinone use (vasodilation medication for post-surgery cardiac support) [111]. However, an increased ratio of 9:13 HODE measured at the start of CPB and at the end of CPB was positively associated with VIS and milirinone use 2–24 h post-surgery, suggesting that increased vasoconstrictive and inflammatory actions of 9-HODE relative to 13-HODE are relevant biomarkers in predicting post-surgical patient outcomes [111]. Supporting the potential vasoconstrictive effects of 9-HODE, a cross-sectional study in non-demented people with controlled hypertension (mean age 65 ± 7.1 years) found a positive association between 9-HODE and MRI-based white matter hyperintensity (WMH), and a negative association between 9-HODE and grey matter volume [112]. Higher WMH and lower grey matter volume is predictive of cognitive decline in people who are non-demented. Although the findings are associative, the study implicates HODEs and CYP oxylipins as potential therapeutic targets in the prevention of ADRD.

### 8.3. HEPEs

HEPEs are EPA-derived oxylipins, of which 5-, 12-, 15-HEPE are formed by LOX, whereas 18-HEPE is formed by two pathways: the aspirin-acetylated COX2 pathway and the CYP ω-hydroxylase pathway. In addition, 18-HEPE is not only the precursor to RvE1 [25], but also possesses anti-inflammatory actions of its own. Although research on HEPEs is emerging and little is known about the function of many lipids in this class, a handful of in vitro studies have reported anti-inflammatory and neurogenic properties associated with 18-HEPE [25,113,114]. Oxylipin 18-HEPE is associated with inhibiting macrophage-mediated inflammation [114]. In a human hippocampal cell line treated with EPA and DHA, followed by exposure to inflammatory cytokines (interferon beta, IL-6, TNFα), the EPA- and DHA-treated neuronal cells exhibited a reduction in cytokine-induced apoptosis, and n-3 treatment ameliorated blunted neurogenesis [113]. Oxylipins associated with anti-inflammatory effects were EPA LOX products of EPA: 18-HEPE, 5-HEPE, CYP products of EPA: 17,18-EpETE, LOX products of DHA: 4-, 20-HDHA, and CYP products of DHA: 19,20-EpDPE [113]. Among participants with stable coronary artery disease enrolled in randomized open-label study comparing 3.6 g/day EPA + DHA or no n-3 supplementation, those with the highest plasma EPA + DHA index (*N* = 16) and lowest plasma EPA + DHA index (*N* = 15) were compared for levels of specialized pro-resolving mediators (SPMs: EPA and DHA LOX-derived lipoxins, resolvins, protectins, and maresins). Furthermore, the ratio of SPM/LTB4 with atherosclerotic plaque formation at 30 months was evaluated [115]. A high n-3 index was associated with higher levels of two SPMs: EPA-derived RvE1 and DHA-derived Mar-1, and 18-HEPE (RvE1 precursor); a low n-3 index was associated with low SPMs; and a low ratio of 18-HEPE + RvE1 to LTB4, an AA-derived proinflammatory oxylipin, was associated with significant atherosclerotic plaque formation as measured by coronary computed tomographic angiography [115]. In addition, a subset of participants with high plasma n-3 levels and a low ratio of 18-HEPE + RvE1 to LTB4 (*N* = 5) had significant plaque formation; conversely, those with a high ratio of 18-HEPE + RvE1 to LTB4 (*N* = 11) exhibited significant plaque regression. It is unclear why five subjects with high plasma n-3 had low SPM/LTB4. The authors conclude that low SPM:LTB4 and specifically, a low 18-HEPE + RvE1 to LTB4 ratio are potential risk factors in coronary artery disease.

Very little is known about 12-HEPE (Figure 3A, Supplementary Table S1). One in vitro study reported the mild inhibition of platelet aggregation [116], and a cross-sectional study in people with hypertension without dementia reported an association of increased plasma levels of 12-HEPE with better executive function performance (Trails-B) [112]. Similarly to 18-HEPE (Figure 3A, Supplementary Table S1), this EPA-derived oxylipin may have protective effects.

### 8.4. CYP and sEH

Cytochrome P40 enzymes convert PUFAs to epoxides that are generally thought to be anti-inflammatory, vasodilatory, and neuroprotective. These effects are enhanced in studies that incorporate the inhibition of sEH, which converts epoxides to diols (Figure 3A,B). sEH activity is often associated with a loss of protective effects from epoxides and/or an increase in detrimental effects [117,118]. However, as mentioned in Section 4, sEH is also suggested to have beneficial roles in some animal studies, depending on the type and sub-cellular localization.

## 9. Oxylipins Altered in ADRD Risk and ADRD Conditions

**Figure 4:**
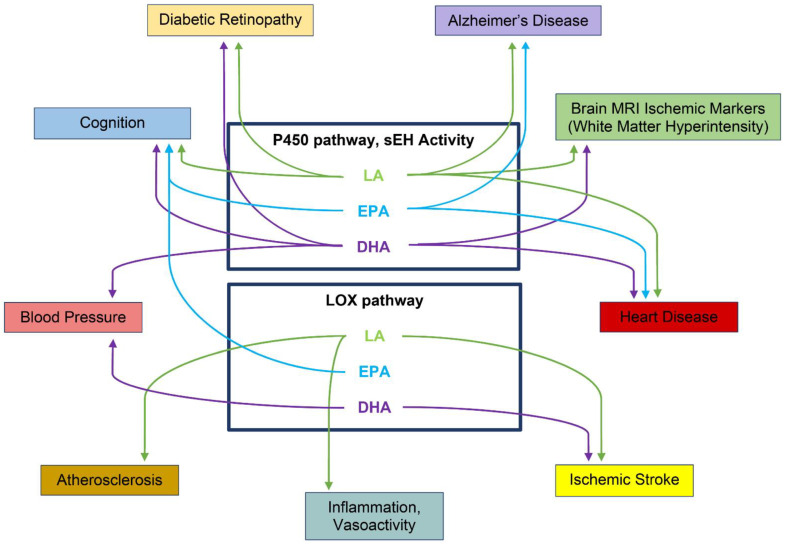
Schematic diagram of oxylipins and associated clinical outcomes. Highlights of the major oxidation pathways for linoleic acid (LA), eicosapentaenoic acid (EPA), and docosahexaenoic acid (DHA) that include cytochrome P450 (P450 pathway)-soluble epoxide hydrolase (sEH), and lipoxygenase (LOX pathway) that are associated with clinical outcomes associated with ADRD and ADRD risk listed in Table 1. First author and references included for clinical outcomes: Diabetic Retinopathy, Zhao et al. [124], Hu et al. [59]; Alzheimer’s Disease, Borkowski et al. [126]; Brain MRI Ischemic Markers (White Matter Hyperintensity), Yu et al. [122], Shinto et al. [112]; Heart Disease, Zhang et al. [121], Kim-Campbell et al. [111]; Ischemic stroke, Szczuko et al. [110]; Inflammation, Vasoactivity, Kim-Campbell et al. [111]; Atherosclerosis, Kuhn et al. [108], Waddington et al. [109], Shibata et al. [105]; Blood Pressure, Caligiuri et al. [119]; Cognition, Yu et al. [122], Shinto et al. [112], Borkowski et al. [125].

A number of studies have demonstrated altered oxylipin composition in ADRD and conditions that increase the risk for ADRD, as described below and summarized in Table 1 and Figure 4.

### 9.1. Cardiovascular

A prospective observational multi-center study in 479 patients with systolic heart failure who received implantable cardioverter–defibrillators (ICDs) evaluated the association of serum oxylipins with ICD shock for ventricular arrhythmias and mortality. Five of the six oxylipins associated with ICD shock suggested increased sEH activity because they were diols, and included: EPA-derived 17,18-DiHETE; DHA-derived 19,20-DiHDPA; AA-derived 5,6-DHET; AA-derived 8,9-DHET; and LA-derived 9,10-DiHOME [121]. The ratio of four of these sEH oxylipins to their precursor PUFAs were associated with mortality (17,18-DiHETE:EPA, 5,6-DHET:AA, 8,9-DHET:AA, and 9,10-DiHOME:LA) [121]. Indeed, the majority of oxylipins associated with either ventricular fibrillation or mortality were products of sEH activity.

### 9.2. Diabetic Retinopathy (DR)

In diabetic retinopathy (DR), increased vascular permeability, decreased microvascular perfusion, and decreased pericyte density are characteristic in disease progression. One study evaluating the effects of sEH activity on vascular permeability in DR involved human DR retinal and vitreous humor samples, as well as a mouse model, to measure sEH activity, CYP-dependent DHA-derived oxylipins, and pericyte loss [59]. In post-mortem retinal samples from patients with mild DR (*N* = 7), severe DR (*N* = 6), and non-DR (*N* = 6), DR severity was positively associated with sEH activity. Comparing vitreous humor from DR (*N* = 17) and non-DR patient (*N* = 14) samples, DR patients had higher levels of DHA-derived 19,20-EpDPE and higher levels of its sEH product 19,20-DiHDPA [59]. Proliferative diabetic retinopathy (PDR) is an advanced and severe form of DR, where long term inflammation and ischemia contribute to retinal neovascularization, retinal detachment, and blindness. Common risk factors for PDR include hyperglycemia, hypertension, and hyperlipidemia [124]. When comparing vitreous humor in patients with PDR (*N* = 41) and controls (*N* = 21), four fatty acids (EPA, DHA, AA, and adrenic acid) and three CYP-derived oxylipins were identified as potential biomarkers distinguishing PDR from controls [124]. The three oxylipins included CYP and sEH derivatives of LA: 12,13-EpOME and 9,10-DiHOME, and a CYP derivative of DHA: 19,20-EpDPE [124].

### 9.3. Hypertension

A randomized controlled trial that evaluated the antihypertensive effects of encapsulated ground flaxseeds versus placebo capsules in participants with peripheral arterial disease (75% hypertensive) found that, after 6 months, systolic and diastolic blood pressure in the flaxseed group were significantly lower compared with the control group [119,120]. Flaxseeds contain a high amount of n-3 ALA, and one of the study aims was to evaluate flaxseed effects on oxylipins. In the flaxseed group, lower levels of sEH-derived oxylipins from DHA, AA, and LA were observed, including sEH-derived 19,20-DiHDPA (from DHA), 5,6-, 8,9-, 11,12- and 14,15-DHETs (from AA), and 9,10- and 11,12-DiHOME (from LA). In addition, CYP hydroxylase-derived 20-HDoHE from DHA was decreased, and LOX-derived HDoHE was increased. Lower total sEH-derived oylipins were associated with lower systolic blood pressure in the flaxseed group [119].

### 9.4. Vascular Cognitive Impairment and Dementia (VCID)

Cerebrovascular disease involves brain ischemia from vessel stenosis, thrombosis, hemorrhage, or embolism, and is a common cause of stroke as well as being significant contributor to VCI. With advancing age, small-vessel ischemic disease (SVID) can be visualized by T2-weighted brain MRI as white matter hyperintensities (WMHs), and greater WMH burden is associated with cognitive decline and increased risk of ADRD [127,128,129]. A study that compared oxylipin sEH activity in patients with extensive subcortical SVID, defined by a high WMH burden, and patients with minimal SVID (lower WMH burden) found that patients with extensive SVID had higher serum levels of LA-derived 12,13-DiHOME, and elevated sEH activity for LA-derived 12,13-DiHOME/12,13-EpOME and 9,10-DiHOME/9,10-EpOME ratios (serum diol/epoxy ratios were used as a measure of sEH activity) [122]. In addition, LA-derived 12,13-DiHOME:12,13-EpOME ratio (i.e., sEH activity) was associated with poorer executive function [122]. Another cross-sectional study in people with controlled hypertension (mean age 65 ± 7.1 years) without dementia showed that increased plasma sEH activity (ratio of plasma diol/epoxide) was associated with higher WMH burden and/or poorer executive function performance [112]. A higher AA-derived 14,15-DHET/14,15-EET ratio was associated with increased WMH burden; a higher LA-derived 9,10-DiHOME:9,10-EpOME ratio was associated with increased WMH burden and poorer executive function performance; and a higher DHA-derived 19,20-DiHDPA:19,20-EpDPE ratio was associated with poorer executive function performance. One DHA-derived CYP epoxide, 16,17-EpDPE, was associated with lower WMH burden [112]. In the same cohort, higher DHA-derived 16,17-EpDPE was associated with increased fractional anisotropy (FA) measured by 3 Tesla MRI diffusion tensor imaging, suggesting greater white matter structural integrity, within the inferior frontal–occipital fasciculus, cingulate, and anterior thalamic radiations, whereas a higher DHA-derived 19,20-DiHDPA:19,20-EpDPE ratio was associated with lower FA in the same tracts [123]. Reports from these MRI studies bolster the hypothesis that epoxides may serve to provide neurovascular protection, while the conversion of epoxides to diols may lead to a loss of protection or even detrimental effects [112,122,123].

### 9.5. Mild Cognitive Impairment (MCI) and Alzheimer’s Disease (AD)

One study that used biobanked serum samples (*N* = 132, 85% cognitively normal and 15% MCI) from two ongoing longitudinal cohorts of aging and AD populations, The Religious Order Study and The Rush Aging and Memory study, was able to identify levels of specific oxylipins and bile acids that correlated with fasting or non-fasting states which were associated with cognitive performance. Furthermore, they developed a useful tool to estimate sample fasting status, if unknown [125]. In fasting samples, higher sEH activities from LA-derived 12,13-DiHOME/12,13-EpOME, EPA-derived 14,15-DiHETE, and DHA-derived 19,20-DiHDPA were associated with poorer performance in perceptual speed (digit symbol modality test); in non-fasting samples, higher LA, EPA, DHA, and EPA LOX-derived 15-HEPE levels were associated with better performance on perceptual speed [125]. If fasting state of sample was unknown, low levels of LA CYP 12,13-EpOME, a conjugated bile acid, glycochenodeoxycholic acid (GCDCA), and elevated levels of glycine-conjugated oleic acid were predictive of fasting [125]. Using biobanked samples from the Emory Goizueta Aging and Alzheimer’s Disease Research Center, another study compared differences in oxylipins between AD and cognitively normal participants in CSF (AD *N* = 150, control *N* = 139) and in plasma (AD *N* = 148, control *N* = 133) [126]. In CSF, AD samples showed higher CYP 9,10-,12–13-EpOME from LA and their corresponding diols 9,10-, 12,13-DiHOMEs, and lower CYP sEH-derived 17,18-DiHETE from EPA. In plasma, AD samples showed higher EPA CYP sEH-derived diol 17,18-DiHETE, and lower EPA-derived 5-, 12-HEPE and DHA-derived 4-, 14-HDoHE. Oxylipins included in models that were predictive of AD included LA CYP-derived 12,13-DiHOME/12,13-EpOME (indicating sEH activity) and AA CYP-derived diol 14,15-DHET [126]. The authors conclude that the observations support the involvement of sEH activity in AD and, because oxylipins modulate vascular tone, the data are supportive of vascular dysfunction as an AD risk factor [126].

## 10. Summary and Future Directions

Long-chain PUFAs have been studied for over 30 years in ADRD risk conditions that include hypertension, cardiovascular disease, type 2 diabetes, and in AD. To date, omega-3 PUFAs evaluated in AD and AD prevention clinical trials have shown no clear benefit. PUFAs sequestered in phospholipid membranes in peripheral and brain tissues are released and oxidized to form oxylipins that have potent bioactive effects, and have been understudied in ADRD. Specific oxylipins that may be relevant to ADRD due to their effects on the vasculature include: LOX-dependent LA-derived HODEs; EPA-derived HEPEs; CYP-dependent LA-derived epoxides 9,10- and 12,13-EpOMEs; EPA-derived epoxide 17,18-EpETE; DHA-derived epoxides 16,17- and 19,20-EpDPE; and CYP sEH-dependent LA-derived 9,10- and 12,13-DiHOMEs; EPA-derived 17,18-DiHETE; and DHA-derived 16,17-EpDPE, 19,20-EpDPE, and 19,20-DiHDPA. Future research focused more specifically on the effects of sEH activity on vascular tone, neurovascular coupling, neuroinflammation, cognitive performance, and vascular brain injury, and relationships between these distinct outcome measures using animal models, and in vivo MRI techniques in both animal and human studies, are warranted. Integrating MRI with plasma biomarker data and incorporating information regarding genetic and dietary heterogeneity will allow for a better understanding regarding the role of specific oxylipins in regulating cerebrovascular health, which is highly relevant for refining strategies to treat and even prevent ADRD using dietary interventions and dietary supplements. More studies performing in-depth pharmacognostical analyses of the types of PUFAs in different dietary sources will further improve the design and efficacy of such interventions.

## Figures and Tables

**Table 1:** Summary table of oxylipin studies involving human subjects.

Study	Population	Methods	Findings	Conclusions
Kuhn et al. (1997) [108]	Advanced atheroma from patients undergoing carotid endarterectomy, Berlin samples (*N* = 17), age 50–70 years Young human lesions obtained from PDAY study samples (*N* = 19) (Wissler 1994), age 15–34 years	Advanced atheroma samples, carotid artery Post-mortem atherosclerotic samples	LA LOX-derived 13S-HODE identified as major oxygenation product of LDL from atherosclerotic tissue samples Advanced atheroma samples: S/R-ratio of 13-HODE was 1:1 HODE/LA ratio 0.2–3.2% Young human lesions: S/R-ratio of 13-HODE was 54:46 HODE/LA ratio 0.05–0.6%	LOX activity is enzymatically active in young human lesions; may be import for early atherogenesis
Waddington et al. (2003) [109]	Patients undergoing carotid endarterectomy, age 70 years, Royal Perth Hospital, Australia Recent TIA or stroke (*N* = 29) Asymptomatic stenosis (*N* = 17)	Atherosclerotic plaques, carotid artery Plaque severity, pathological classification	Component of all plaque types: LA-derived 9- and 13-HODES AA-derived 15- and 11-HETE No difference in oxylipins according to plaque histopathology No difference in oxylipins between symptomatic and asymptomatic groups LA oxidation products higher in alcohol consumers AA oxidation products higher in symptomatic PVD	Did not identify FA oxidation products associated with plaque instability and symptomatic CVD
Shibata et al. (2009) [105]	Patients undergoing carotid endarterectomy (*N* = 6)	Atherosclerotic plaques, carotid artery	13(R)-HODE staining on VEC, macrophages and migrating VSMC 13(R)-HODE immunoreactivity more intense in the macrophage-enriched plaques Colocalization of 13(R)-HODE with OxPC, PPAR (gamma), and CD36 in atherosclerotic lesions	13(R)-HODE, non-enzymatically formed in the presence of oxidative stress, is a major component of atherosclerotic lesions
Caligiuri et al. (2014) [119]	FlaxPAD participants (Leyva 2011) Original cohort (*N* = 110), age 66.4 years *N* = 98 at baseline with oxylipin data *N* = 76 at 6 months with oxylipin data Flaxseed-enriched vs. control diet	Plasma Systolic BP Diastolic BP	No difference in baseline oxylipins between intervention and control diet groups Flaxseed vs. control group: DHA CYP sEH-derived 19,20-DiHDPA decrease DHA CYP hydroxylase-derived 20-HDoHE decrease DHA LOX-derived 4-HDoHE increase AA CYP sEH-derived 5,6-, 8,9-, 11,12-, 14,15-DHET decrease LA CYP sEH-derived 9,10- and 12,13-DiHOME decrease Lower total sEH-derived oxylipin associated with lower SBP Higher concentration Alpha-linolenic acid associated with decreased sEH activity	Flaxseed LA may inhibit sEH activity, resulting in altered oxylipin concentrations that contribute to blood pressure reduction in patients with PAD
Caligiuri et al. (2016) [120]	FlaxPAD participants (Leyva 2011) Original cohort (*N* = 110), age 66.4 years *N* = 62, baseline and 6 months *N* = 41/62 with HTN diagnosis	Plasma Radial tonometry (cBP)	Flaxseed vs. control group: cDBP decreased at 6 and 12 months (*N* = 62) cSPB decreased at 12 months (*N* = 41 with HTN) Every 1 nmol/L increase in AA CYP hydrolase-derived 16-HETE increased odds of higher central systolic and diastolic BP by 12- and 9-fold, respectively Every 1 nmol/L increase in AA CYP sEH-derived 5,6-DHET increased the odds of higher cBP by 9-fold AA CYP sEH-derived 11,12-DHET and AA CYP hydrolase-derived 16-HETE associated with greater cMAP	Study provides support for oxylipins as therapeutic targets in HTN
Zhang et al. (2016) [121]	PROSE-ICD study (*N* = 479), age 60, patients with heart failure and implanted defibrillator	serum	Oxylipins associated with increased risk of ICD shock: EPA-derived 17–18-DiHETE DHA-derived 19,20-DiHDPA AA CYP sEH-derived 5,6-DHET AA CYP sEH-derived 8,9-DiHET LA CYP sEH-derived 9,10-DiHOME PGF 1-alpha Oxylipin-to-precursor ratios associated with mortality: 15S-HEPE/EPA 17,18-DiHETE/EPA 19,20-DiHDPA/DHA 5S-HEPE/EPA	Novel oxylipin markers identified associated with ventricular fibrillation and all-cause mortality in patients with heart failure
Hu et al. (2017) [59]	Wilmer Eye Institute, Johns Hopkins *N* = 6, non-DR, age 75.7 ± 3.1 years *N* = 7, mild DR, age 57.2 ± 13.0 years *N* = 6, severe DR, age 62.2 ± 11.8 years	Post-mortem retinal samples	Increased sEH expression with disease severity in those with DR	DR severity associated with increased sEH expression
Hu et al. 2017 [59]	Henan Eye Institute, Henan, China *N* = 17 DR, age 57.0 ± 9.9 years *N* = 14 non-DM macular disease, age 58.9 ± 8.0 years	Vitreous humor samples from patients undergoing vitrectomy	Greater DHA CYP sEH substrate 19,20-EpDPE and sEH product 19,20-DiHDPA, in diabetic retinopathy samples	DR associated with increased DHA sEH-derived oxylipin
Yu et al. (2019) [122]	Patients with recent TIA and high WMH (*N* = 29), age 71.8 years Healthy elderly controls with low WMH (*N* = 25), age 71.7 years MMSE > 19, no cortical stroke or Alzheimer’s disease diagnosis	Serum 3T MRI Psychometric testing	High WMH group vs. low WMH group: LA CYP sEH-derived 12,13-DiHOME and elevated 12,13-DiHOME/12,13-EpOME ratio elevated 9,10-DiHOME/9,10-EpOME ratio elevated 12,13-DiHOME/12,13-EpOME ratio associated with poorer performance on composite test of executive function PV WMH explained 37% of the effect of the 12,13-DiHOME/12,13-EpOME ratio on executive function	Oxylipin changes associated with higher sEH activity may be use as markers for age-related VCI
Szczuko et al. (2020) [110]	Patient with ischemic stroke (*N* = 75), District Hospital, Poland Control Group (*N* = 35), University of the Third Age, Poland	Fasting plasma	Ischemic vs. control group: AA COX-derived PGE_2_ higher AA LOX-derived 15-HETE higher AA CYP hydroxylase-derived 16-HETE lower AA LOX-derived 5-HETE and 5 oxo ETE lower DHA LOX-derived RvD1 lower EPA COX2-derived 18-HEPE higher LA LOX-derived 9-HODE and 13-HODE lower	Study confirms the involvement of FFA-derivative mediators of inflammation associated with ischemic stroke
Shinto et al. (2020) [112]	Community-based sample, Portland, OR *N* = 37, controlled HTN, age 65.6 ± 7.1 years, no dementia	Fasting plasma 3T MRI	LA LOX-derived 9-HODE associated with greater WMH and reduced GM volume LA CYP sEH-derived 9,10-DiHOME/EpOME ratio associated with increased WMH and poorer performance on Trails-B AA CYP sEH-derived 14,15-DHET/EET ratio associated with increased WMH DHA CYP sEH-derived 19,20-DiHDPA/EpDPE ratio associated with increased WMH and poorer performance on Trails B DHA CYP-derived 16,17-EpDPE associated with lower WMH Parent compounds LA and DHA had no significant relationship with Trails B or MRI outcomes EPA LOX-derived 12-HEPE associated with better performance on Trails B	Specific oxylipin products, and not parent compounds LA or DHA, are associated with MRI and cognitive markers of dementia risk, including that from VCI, in young–old individuals
Silbert et al. (2020) [123]	Community-based sample, Portland, OR *N* = 37, controlled HTN, age 65.6 ± 7.1 years, no dementia (*N* = 36 with DTI)	Fasting plasma 3T MRI TBSS DTI	DHA CYP-derived 16,17-EpDPE associated with: Increased FA within the IFOF, cingulate and ATR tracts DHA CYP sEH-derived 19,20-DiHDPA/EpDPE associated with: Decreased FA within the IFOF, cingulate and ATR tracts Decreased FA within the SLF, ILF, and CST DHA parent compound was not related to DTI measures	DHA-derived oxylipins, not DHA are associated with WM integrity in hypertensive young-old individuals at risk for VCI
Kim-Campbell et al. (2020) [111]	Children undergoing CPB surgery at the Children’s Hospital of Pittsburg *N* = 34, age 2.5 years (0.6–12.0)	Blood collected start and end of CPB PHb, plasma oxylipins, VIS	Difference from CPB start to CPB end: No change in LA 9-HODE and 13-HODE levels increased 9:13-HODE ratio decreased Higher 9:13-HODE ratio at CPB start and end associated with VIS and milrinone use Higher 9:13 HODE ratio at CPB start associated with WBC increase	9:13-HODE ratio is a relevant biomarker of vascular tension and inflammation in a pediatric population undergoing CPB surgery
Welty et al. (2021) [115]	Subset of participants in HEARTS trial; EPA + DHA treatment in patients with CAD (Alfaddagh 2017), BIDMC, Boston, MA 31 subjects with the highest (*N* = 16) and lowest plasma omega-3 fatty acid index EPA + DHA/total fatty acids (*N* = 15) at 30-month follow-up. mean age in both groups, around 63 years	Plasma omega-3 fatty acid index (EPA + DHA/total fatty acid level), and oxylipins Coronary plaque volume measured using CCTA	High omega-3 fatty acid index associated with: Higher EPA-derived 18-HEPE and Resolvin E1 Higher levels DHA LOX-derived Maresin 1 Low omega-3 fatty acid index associated with: lower EPA COX2-derived Resolvin E1 lower DHA LOX-derived Maresin 1 lower (18-HEPE + resolvin E1)/LTB4 ratio increased progression of plaque formation Those with high omega-3 fatty acid index and low (18-HEPE and resolvin E1)/LTB4 had significant plaque formation (*N* = 5) Those with high (18-HEPE + resolvin E1)/LTB4 had significant plaque regression (*N* = 11)	A low ratio of specialized pro-resolving mediators to proinflammatory mediator, specifically (18-HEPE + RvE1)/LTB4, is a potential novel risk factor associated with coronary plaque progression in patients with CAD
Zhao et al. (2022) [124]	Department of Ophthalmology, Second Xiangya Hospital of Central South University Vitreous humor samples from patients with PDR (*N* = 41) and non-diabetic controls (*N* = 22)	Vitreous humor samples obtained during vitrectomy	Oxylipins with AUC ≥ 0.8; Potential biomarkers distinguishing PDR from controls: EPA, DHA, AA, Adrenic Acid LA CYP sEH-derived 9,10-DiHOME LA CYP-derived 12,13-EpOME DHA CYP-derived 19,20-EpDPE	LOX- and CYP-products were the most affected oxylipins, altered balance between inflammatory and anti-inflammatory oxylipins in PDR
Borkowski et al. (2021) [125]	Participants from the RUSH ROS and MAP (*N* = 198). Age 78.2 (7.2), 22% male, 95% white, NCI and MCI. 212 serum samples: fasted (*N* = 59), non-fasted (*N* = 80), unknown if fasted (*N* = 73);	Fasted and non-fasted oxylipins, endocannabinoids, bile acids and steroids Cognitive battery	Associations with worse processing speed, fasted: Higher LA CYP sEH-derived 12,13-DiHOME/12,13EpOME Higher EPA CYP sEH-derived diol 14,15- and 17,18-DiHETE Higher DHA CYP sEH-derived diol 19,20-DiHDPA Higher sum of the omega-3 diols Lower AA Sum of EPA and DHA diols most predictive Associations with worse processing speed, non-fasted: Lower LA, EPA, DHA, and EPA LOX-derived 15-HEPE Fasting sample used to predict a high probability of fasted state if state unknown in sample: low levels LA CYP 12,13 EpOME low GCDCA (conjugated bile acid) ochenodeoxycholic acid (GCDCA) elevated levels of the glycine-conjugated oleic acid	Omega-3 sEH (diols) are predictive of poorer perceptual speed performance in samples obtained in fasting state; developed tool to estimate is sample is fasted, if unknown
Borkowski et al. (2021) [126]	Emory Healthy Brain Study (Emory ADRC) and Emory Cognitive Neurology Clinic CSF: AD, *N* = 150, age 68.2 (1.3), 47% male, 70% ApoE4, control, *N* = 139, age 65 (1.4), 28% male, 25% ApoE4 Plasma: AD, *N* = 148, age 68 (1.4), 48% male, 70% ApoE4, control, *N* = 133 age 66 (1.5), 27% male, 24% ApoE4	Oxylipins, endocannabinoids, bile acids, steroids. Estimated fasted samples [125] MoCA	Estimated fasting plasma; lower in AD vs. Controls: EPA and DHA LOX-derived oxylipins EPA-derived 5-, 9-, 12-HEPE; DHA-derived 4-, 14-HDoHE Estimated fasting plasma; higher in AD vs. Controls: EPA CYP sEH-derived diol 17,18-DiHETE CSF: higher in AD vs. Control: LA CYP 9,10-,12–13-EpOME and corresponding sEH-derived DiHOMEs CSF: lower in AD vs. Control: EPA and DHA; EPA CYP sEH-derived 14, 15-DiHETE Predictive model in plasma strongest, LA CYP 12,13-DiHOME/12,13-EpOME (indicating sEH activity) and AA CYP diol 14,15-DHET were included in model.	AD is associated with elevations in fasting plasma and CSF CYP sEH activity, supporting vascular dysfunction as a factor in AD

For PUFA derivative, oxylipin, and enzymatic pathway abbreviations, see Table 1. ADRC = Alzheimer’s Disease Research Center, ATR = anterior thalamic radiation, BIDMC = Beth Israel Deaconess Medical Center, CAD = coronary artery disease, cBP = central blood pressure, CCTA = coronary computed tomographic angiography, cDBP = central diastolic blood pressure, CI = cognitively intact, cMAP = central mean arterial pressure, CPB = cardiopulmonary bypass, CST = corticospinal tract, CVD = cerebrovascular disease, DM = diabetes mellites, DR = diabetic retinopathy, FFA = free fatty acid, FlaxPAD = Flaxseed for Peripheral Arterial Disease, GM = gray matter, HEARTS = Slowing HEART diSease with lifestyle and omega-3 fatty acids, HPLC = high-performance liquid chromatography, HTN = hypertension, IFOF = inferior frontal–occipital fasciculus, ILF = inferior longitudinal fasciculus, LTB4 = leukotriene B4, MAP = Memory and Aging Project, MCI = mild cognitive impairment, NCI = no cognitive impairment, OADRC = Oregon Alzheimer’s Disease Research Center, OBAS = Oregon Brain Aging Study, OxPC = oxidized phosphatidylcholine, PDAY = pathological determinants of atherosclerosis in youth, PGF1a = prostaglandin f-1 alpha, PHb = plasma hemoglobin, PPAR(gamma) = peroxisome proliferator-activated receptor gamma, PROSE-ICD = Prospective Observational Study of Implantable Cardioverter-Defibrillators (PROSE-ICD), PV = periventricular, PVD = peripheral vascular disease, ROS = Religious Orders Study, SBP = systolic blood pressure, SLF = superior longitudinal fasciculus, TIA = transient ischemic attack, WMH = white matter hyperintensity, VEC = vascular endothelial cells, VCI = vascular cognitive impairment, VIS = vasoactive inotropic score, VSMC = vascular smooth muscle cells.

## Supplementary Materials

The following supporting information can be downloaded at: https://www.mdpi.com/article/10.3390/metabo12090826/s1, Table S1: Oxylipin names and abbreviations.
