# Supplementary material for: A Review of Oxylipins in Alzheimer’s Disease and Related Dementias (ADRD): Potential Therapeutic Targets for the Modulation of Vascular Tone and Inflammation
Source: Metabolites. 2022 Sep 1;12(9):826. doi: 10.3390/metabo12090826 (PMC9501361; doi:10.3390/metabo12090826)
Supplement: Supplementary file 1 [file metabolites-12-00826-s001.zip › metabolites-1817989-supplementary.pdf]

Supplementary Table S1. Oxylipin names and abbreviations.

Abbreviation	PUFA Derivative	PUFA Type	Enzymatic Pathway	Name
$PGA_{2,}$, $PGB_{2,}$ $PGE_{2,}$	AA	n-6	COX	Series 2 prostaglandin- A, B, E
$TxA_{2,}$, TxB_2	AA	n-6	COX	Series 2 thromboxane- A, B
5-, 8-, 9-,11-,<br>12-, 15-, HpETE	AA	n-6	LOX	5-, 8-, 9--,11-, 12-, 15-hydroxperoxy-eicosatetraenoic acid
5-, 8-, 9-,11-,<br>12-, 15-, HETE	AA	n-6	LOX	5-, 8-, 9--,11-, 12-, 15-hydroxy-eicosatetraenoic acid,
LTB_4, LTC_4, LTD_4,<br>LTE_4, $LTF_{4,}$ LxB_4	AA	n-6	LOX	Series 4 Leukotriene- A-F; Series 4 Lipoxin- A,B
5, 6-, 8,9-, 11,12-<br>14,15-, EET	AA	n-6	CYP	5,6-, 8,9-, 11,12,<br>14,15-, epoxy-eicosatrienoic acid
5,6-, 8,9-, 11,12,<br>14,15-, DHET	AA	n-6	CYP,sEH	5,6-, 8,9-, 11,12-, 14,15-dihydroxy-eicosatrienoic acid
16-,17-,18-,19-,20-HETE	AA	n-6	CYP, ω-hydroxylase	16-,17-,18-,19-,20--hydroxyeicosatetraenoic acid
9-HODE, 13-HODE	LA	n-6	LOX	9-, 13- hydroxy-octadecadienoic acid
9,10-, 12,13- EpOME	LA	n-6	CYP	9,10-, 12,13- epoxy-octadecenoic acid
9,10-, 12,13- DiHOME	LA	n-6	CYP,sEH	9,10-, 12,13- dihydroxy-octadecenoic acid
PGD_3, PGE_3<br>PGF_3, $PGJ_{3,}$ TxA_3	EPA	n-3	COX	Series 3 prostaglandin-, D,E,F, J; Series 3 thromboxane A
18-HEPE, RvE1-3	EPA	n-3	COX2*	18-hydroxyeicosapentaenoic acid, resolvin E1-E3
5-, 8-, 9-,11-,<br>12-, 15-, HpEPE	EPA	n-3	LOX	5-, 8-, 9-,11-,<br>12-, 15-hydroperoxy-eicosapentaneoic acid
5-, 8-, 9-,11-,<br>12-, 15- HEPE	EPA	n-3	LOX	5-, 8-, 9-,11-,<br>12-, 15- hydroxyeicosapentaenoic acid

LTBA$_5$, LTB$_5$, LTC$_5$, LTD$_5$, LxA$_5$, Lx	EPA	n-3	LOX	Series 5 leukotrienes A-D; series 5 Lipoxins A, B
5,6-, 8,9-, 11,12-,14,15-17,18- EpETE	EPA	n-3	CYP	5,6-, 8,9-, 11,12-,14,15-17,18- epoxy-eicosatetraenoic acid
5,6-, 8,9-, 11,12-,14,15-17,18- DiHETE	EPA	n-3	CYP,sEH	5,6-, 8,9-, 11,12-,14,15-17,18-dihydroxy-eicosatetraenoic acid
RvD1-4	DHA	n-3	COX2*	Resolvin D1-4
Mar, PD, Rv	DHA	n-3	LOX	Maresins, Protectins, Resolvins
4-,7-,8-,10-,11-,13-HDoHE	DHA	n-3	LOX	4-,7-,8-,10-,11-,13-hydroxydocosahexaenoic acid
7,8-, 10-11, 13,14-16,17-,19,20- EpDPE	DHA	n-3	CYP	7,8-,10-11, 13,14-16,17-19,20- epoxy-docosapentaneoic acid
7,8-, 10-11, 13,14-16,17-,19,20- DiHDPA	DHA	n-3	CYP,sEH	7,8-,10-11, 13,14-16,17-19,20-dihydroxy-docosapentaenoic acid
20-,21-,22-HDoHE	DHA	n-3	CYP, ω-hydroxylase	20-,21-,22-hydroxydocosa-hexaenoic acid

AA, arachidonic acid; LA, linoleic acid; EPA, eicosapentaenoic acid; DHA, docosahexaenoic acid; n-6, omega-6; PUFA, n-3, omega-3 PUFA; COX,cyclooxygenase, *COX2, aspirin-acetylated cyclooxygenase 2; LOX, lipoxygenase; CYP, cytochrome P450; sEH, soluble expoxide hydrolase.
